# Oxidative stress‐induced angiogenesis is mediated by miR‐205‐5p

**DOI:** 10.1111/jcmm.14822

**Published:** 2019-12-21

**Authors:** Maria Oltra, Lorena Vidal‐Gil, Rosa Maisto, Javier Sancho‐Pelluz, Jorge M. Barcia

**Affiliations:** ^1^ Escuela de Doctorado Universidad Católica de Valencia San Vicente Mártir Valencia Spain; ^2^ Neurobiología y Neurofisiología, Facultad de Medicina y Ciencias de la Salud Universidad Católica de Valencia San Vicente Mártir Valencia Spain; ^3^ Centro de Investigación Traslacional San Alberto Magno Universidad Católica de Valencia San Vicente Mártir Valencia Spain; ^4^ Department of Experimental Medicine Università degli studi della Campania Luigi Vanvitelli Napoli Italy

**Keywords:** angiogenesis, microRNAs, retinal pigment epithelial cells

## Abstract

miR‐205‐5p is known to be involved in VEGF‐related angiogenesis and seems to regulate associated cell signalling pathways, such as cell migration, proliferation and apoptosis. Therefore, several studies have focused on the potential role of miR‐205‐5p as an anti‐angiogenic factor. Vascular proliferation is observed in diabetic retinopathy and the ‘wet’ form of age‐related macular degeneration. Today, the most common treatments against these eye‐related diseases are anti‐VEGF therapies. In addition, both AMD and DR are typically associated with oxidative stress; hence, the use of antioxidant agents is accepted as a co‐adjuvant therapy for these patients. According to previous data, ARPE‐19 cells release pro‐angiogenic factors when exposed to oxidative insult, leading to angiogenesis. Matching these data, results reported here, indicate that miR‐205‐5p is modulated by oxidative stress and regulates VEGFA‐angiogenesis. Hence, miR‐205‐5p is proposed as a candidate against eye‐related proliferative diseases.

## 1 INTRODUCTION

MicroRNAs (miRNAs) are non‐coding RNAs 1, 2 able to repress or degrade complementary mRNA of target genes involved in different biological processes including cell proliferation,^3^ cell survival,^4^ inflammation 5 and angiogenesis.^6^ Vascular endothelial growth factor A (VEGFA) is one of the most important angiogenic factors involved in different types of cancer.^7^ In fact, anti‐VEGF therapies are widely used against cancer.^8^, ^9^, ^10^ Actually, several reports indicate a direct role of miR‐205‐5p on VEGFA expression.^7^, ^11^, ^12^, ^13^ It has been observed that miR‐205‐5p directly suppresses expression of VEGFA and reduces cell proliferation‐migration in human glioblastoma cells.^7^ Moreover, An et al. showed that miR‐205‐5p inhibited the VEGF‐PI3K/AKT pathway.^13^ Other studies confirmed that miR‐205‐5p could inhibit the progression of tumour cells, targeting FOXO1^14^ and CBX1,^15^ among others, and promotes LRRK2‐mediated apoptosis.^16^

Enhancement of VEGFA and neovascular overgrowth are behind two associated proliferative retinal disorders: diabetic retinopathy (DR)^17^ and the neovascular—aka ‘wet’ —age‐related macular degeneration (AMD).^18^, ^19^ Even though the significant release of VEGF has been reported several times,^20^, ^21^, ^22^ little is known about the role of miR‐205‐5p in these diseases. VEGFA is released by retinal cells, including the retinal pigment epithelium (RPE).^23^, ^24^ This layer constitutes the blood‐retinal barrier, playing a pivotal role between the bloodstream and photoreceptors.^25^ For several reasons (light, metabolic rate, cellular membrane interactions, etc), RPE is continually exposed to oxidative stress (OS) 26, 27, 28; hence, OS is considered a relevant risk factor for AMD.^27^, ^28^, ^29^ Recent reports point out that OS in RPE cells intensifies the production and release of angiogenic factors, thus increasing the formation of new blood vessels.^20^, ^30^, ^31^ Specifically, OS promotes VEGF production in RPE cells, leading to choroidal neovascularization.^18^, ^32^, ^33^ In agreement with this, miR‐205‐5p overexpression also reduced reactive oxygen species (ROS) by increasing peroxiredoxin 2 levels,^34^ suggesting that miR‐205‐5p could be sensitive to oxidative stimuli. Besides, OS is involved in eye diseases, such as AMD, and VEGFA‐mediated angiogenesis plays a crucial role in the evolution of these diseases.^27^, ^29^, ^35^, ^36^ Indeed, VEGF inhibitors are widely used in both cancer and wet AMD, with beneficial results.^37^, ^38^

The present work focuses on the response of miR‐205‐5p‐VEGFA in ARPE‐19 cells to oxidative challenge as an approach to the potential therapeutic target of eye‐related proliferative disorders.

## 2 MATERIALS AND METHODS

### 2.1 Cell culture

The ARPE‐19 human cell line was obtained from American Type Culture Collection (ATCC). Cells were cultured in Dulbecco's modified Eagle's medium DMEM/F12 (Invitrogen), as previously described.^20^ Cells from passage numbers 11‐30 were used and cultured to 80%‐90% confluence at a starting density of 1 × 10^6^ cells/cm^2^ on different plates depending on the technique. After 2 days, the cells were treated for 24 hours with 600 µmol/L H_2_O_2_ (Scharlau) and 4 mM N‐acetylcysteine (NAC) (Sigma‐Aldrich) using media with 1% of fetal bovine serum (FBS). Cells and supernatant were collected and preserved for further experiments.

Human umbilical vein endothelial cells (HUVEC) were isolated from umbilical veins as previously described.^39^ HUVEC were grown in Endothelial Cell Media supplemented with 20% FBS, penicillin/streptomycin and amphotericin at 37°C and 5% CO_2_.

### 2.2 Determination of intracellular ROS

Intracellular ROS levels were measured using dihydroethidium (DHE; Thermo Fisher Scientific), which is a superoxide indicator. This molecule has a blue fluorescence but, when oxidized to ethidium, it stains DNA red. ARPE‐19 cells were seeded at 6 × 10^3^ cells/well in a 96 well plate. Cells were rinsed with PBS (phosphate‐buffered saline) twice and incubated with 5 μmol/L DHE for 30 min at 37°C and 5% CO_2_. ROS levels were measured by a fluorescence multiplate reader (Victor X5; Perkin Elmer) excited at 518 nm and read at 605 nm.

### 2.3 Mimic and inhibitor transfection

ARPE‐19 cells seeded in a 6 well plate at 60%‐80% confluence were transfected with miR‐205‐5p mirVana^®^ miRNA mimic (Thermo Fisher Scientific) miR‐205‐5p mirVana® miRNA inhibitor and mirVana™ miRNA mimic negative control #1 (Thermo Fisher Scientific), as a negative control of transfection process, at a concentration of 30 pmol (10 µM), using Lipofectamine^®^ RNAiMAX Reagent (Thermo Fisher Scientific) diluted in opti‐MEM^®^ Medium (Thermo Fisher Scientific). After 48 hours of transfection, the cell pellet and the media were collected for further analysis.

The groups were classified as: Control (physiological condition without transfection), Control + mimic miR‐205‐5p (physiological condition and transfection), Control + mimic Control‐ (physiological condition and transfection control), H_2_O_2_ 600 µmol/L (oxidative stress condition), H_2_O_2_ 600 µmol/L + mimic miR‐205‐5p (oxidative stress condition and transfection) and H_2_O_2_ 600 µmol/L + mimic Control‐ (oxidative stress condition and transfection control).

### 2.4 RNA isolation and miRNA expression analysis

The RNA was isolated from ARPE‐19 cells using the miRNeasy Mini Kit (Qiagen) according to the manufacturer's instructions. Total RNA quantity and quality (260/280 absorbance ratio) were assessed using NanoDrop 2000 (Thermo Fisher Scientific). Quantitative real‐time PCR (qRT‐PCR) was used to analyse the miRNA expression profile of the selected miRNAs. For miRNA expression analysis, 100‐300 ng of RNA were retrotranscribed using TaqMan MicroRNA Reverse Transcription Kit (Applied Biosystems) using specific TaqMan RT primers and the thermocycler PeqSTAR 96 Universal Gradient (PeqLAB), with cycles of 16°C/30 min, 42°C/30 min, 85°C/5 min and 4°C/infinity. qRT‐PCR was performed using TaqMan™ microRNA Assays (Thermo Fisher Scientific) with TaqMan Gene Expression Master Mix (Applied Biosystems) and RT‐PCR Roche 234 LighterCycler 480 with the appropriate temperature cycles. Normalization was done with RNU6B snoRNA. Relative expression was calculated as 2^‐ΔΔ^ *^C^* ^t^. For mRNA expression analysis, 1,000 ng of RNA were retrotranscribed with SuperScript III First‐Strand Synthesis System (Life Technologies, Thermo Fisher Scientific) and the thermocycler PeqSTAR 96 Universal Gradient (PeqLAB), with cycles of 65°C/5 min, 4°C/5 min, 55°C/50 min, 85°C/5 min 37°C/20 min and 4°C/infinity. Quantitative real‐time PCR was performed using Sybr Green Supermix (BioRad) and RT‐PCR Roche 234 LighterCycler 480 with the appropriate temperature cycles. Normalization was done with GAPDH. Relative expression was calculated as 2^‐ΔΔCt^.

### 2.5 Analysis of miRNA target genes

In silico analysis of the putative targets of miR‐205‐5p was performed using Target Scan Human (http://www.targetscan.org/vert_72/). We analysed the pathways regulated by the putative targets of miR‐205‐5p with FUNRICH using the gene ontology database. With STRING (https://string-db.org/) we performed the networking of the targets related to angiogenic pathways.

### 2.6 Western blotting

ARPE‐19 cells, treated or control, were collected in RIPA Buffer (Thermo Fisher Scientific) and a protease/phosphatase inhibitor cocktail (Sigma‐Aldrich). Equal amounts of protein (30 µg) were loaded and measured by SDS‐PAGE on 4%‐12% SDS‐Polyacrylamide gel electrophoresis and electro‐blotted onto a polyvinylidene difluoride membrane (PVDF; Millipore) by wet transfer. Membranes were incubated overnight with antibodies against VEGFA (1:200; Santa Cruz Biotechnology) and ß‐actin (1:1,000; Santa Cruz Biotechnology) as loading control. Finally, the membranes were incubated for 2 hours at room temperature with anti‐mouse and anti‐rabbit IgG‐HRP antibodies (Santa Cruz Biotechnology). The visualization was performed with ECL (Pierce, Thermo Fisher Scientific) and detected with Image Quant LAS‐100 mini (GE Healthcare). Protein levels were quantified by densitometry using ImageJ software (NIH).

### 2.7 Vasculogenesis assay

Vasculogenesis was analysed in normal Matrigel (Becton Dickinson) as previously described.^39^ Briefly, 8 × 10^4^ HUVEC cells/well in a 96 well plate were seeded and treated or not with ARPE‐19 cells media for 5 hours. The different conditions were determined by the media used; control media, 600 µmol/L H_2_O_2_ media, miR‐205‐5p mimic‐transfected control media and miR‐205‐5p mimic‐transfected‐600 µmol/L H_2_O_2_ media from ARPE‐19 cells. Matrigel was diluted with DMEM/F12, FBS‐free and allowed to solidify overnight at 37°C. Pictures were taken with a CKX41 inverted microscope (Olympus) and images recorded by an DP74 digital camera (Olympus). The images were analysed with Image J using the Angiogenesis Analyzer.

### 2.8 Statistical analysis

Results of each experiment are presented as mean ± SEM. Statistical significance was set at 0.05 by using *t* test and 2‐way‐ANOVA.

## 3 RESULTS

### 3.1 Oxidative stress down‐regulates miR‐205‐5p expression

**Figure 1:**
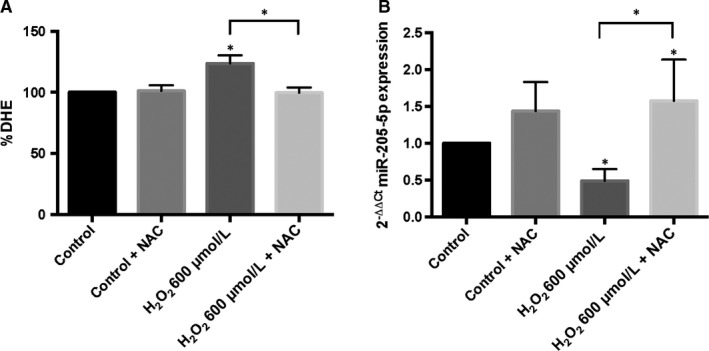
Oxidative stress regulates miR‐205‐5p expression. Superoxide anions were measured by DHE after H_2_O_2_ and NAC exposure (A). ARPE‐19 miR‐205‐5p expression was analysed by qRT‐PCR (B). Values are expressed as mean ± SEM (n = 3). Statistically significant differences were set at *P < .05

As previously observed, ARPE‐19 cells resulted in a significant ROS production after 600 µM H_2_O_2_ exposure.^40^, ^41^ This phenomenon was normalized by N‐acetylcysteine (NAC) (Figure 1A). Significantly reduced miR‐205‐5p levels were observed in ARPE‐19 cells after H_2_O_2_ exposure compared with control cells (Figure 1B). In order to assess whether this increase is oxidative stress‐dependent, the antioxidant NAC (4 mmol/L) was added. NAC normalized the miR‐205‐5p levels reduced by H_2_O_2_. No significant changes in miR‐205‐5p expression levels were found in control ARPE‐19 cells after NAC exposure (Figure 1B).

### 3.2 miR‐205‐5p targets are related to vascular processes

**Figure 2:**
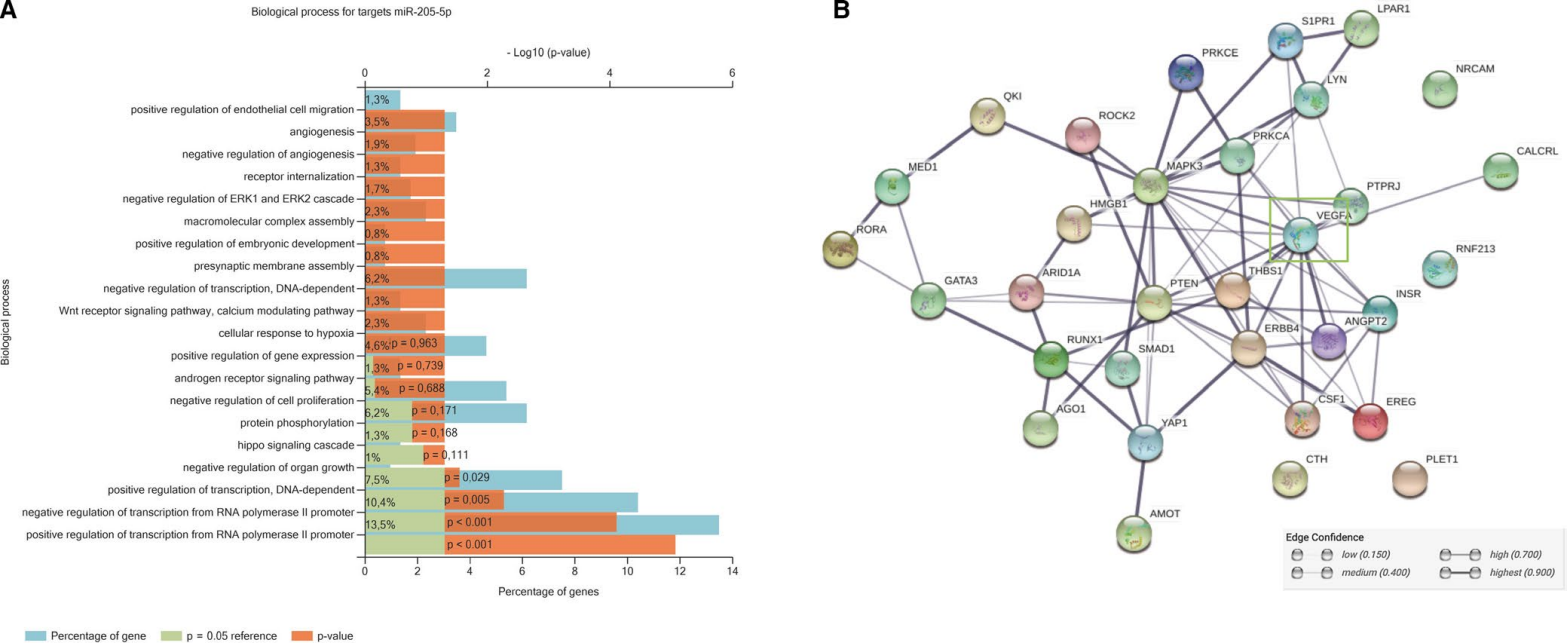
miR‐205‐5p protein target‐related pathways. Top 20 biological processes regulated by miR‐205‐5p predicted targets, using FUNRICH and the gene ontology database (A). Interactional Network of miR‐205‐5p angiogenic related targets (B)

Potential biological processes regulated by miR‐205‐5p were assessed using in silico analysis with TargetScan (http://www.targetscan.org/vert_72/). Actually, 592 putative mRNA targets were found. To analyse the biological pathways and networks regulated by the predicted targets of miR‐205‐5p, these 592 mRNAs were entered into FUNRICH software (version 3.1.3). Functional enrichment analysis showed more than 2800 biological processes regulated by at least one mRNA target of miR‐205‐5p. The top 20 biological processes regulated by miR‐205‐5p are shown in Figure 2A. After selecting angiogenic and vasculogenic related processes, 32 mRNA predicted targets were found according to their functional protein association network, using STRING (https://string-db.org/) (Figure 2B and File S1).

### 3.3 VEGFA mRNA is a direct target of miR‐205‐5p

**Figure 3:**
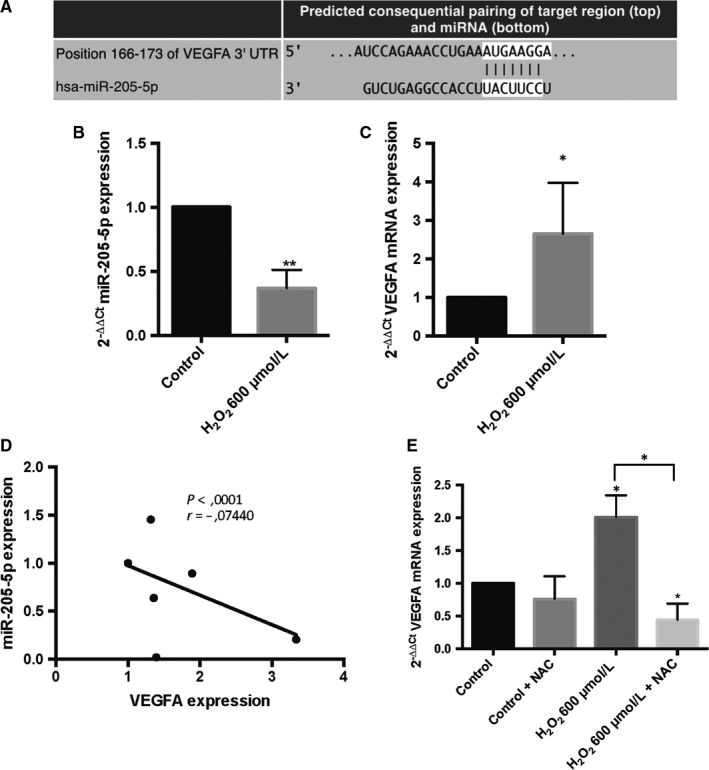
VEGFA is predicted as direct target of miR‐205‐5p. Predicted binding site of miR‐205‐5p with the 3’UTR mRNA VEGFA (A). qRT‐PCR H_2_O_2_ exposure of miR‐205‐5p expression (B) and VEGFA mRNA expression (C). Spearman's correlation between miR‐205‐5p and VEGFA mRNA (D). qRT‐PCR VEGFA mRNA expression after NAC exposure (E). Values are expressed as mean ± SEM (n = 3). Statistically significant differences were set at *P < .05 and **P < .01

Consistently with previous results, in silico analysis showed that VEGFA mRNA is a predicted target for miR‐205‐5p (Figure 2B). Target Scan predicted a 3’‐UTR binding site for VEGFA mRNA and miR‐205‐5p (Figure 3A). In order to confirm the relation between VEGFA mRNA and miR‐205‐5p, VEGFA mRNA expression was analysed after H_2_O_2_ treatment. We observed that VEGFA mRNA levels were up‐regulated after H_2_O_2_ exposure (Figure 3C), indicating a negative correlation with miR‐205‐5p levels (Figure 3B and 3D). Matching with the aforementioned results on miR‐205‐5p levels, VEGFA mRNA levels were also normalized by NAC exposure (Figure 3E).

### 3.4 VEGFA expression is regulated by miR‐205‐5p

**Figure 4:**
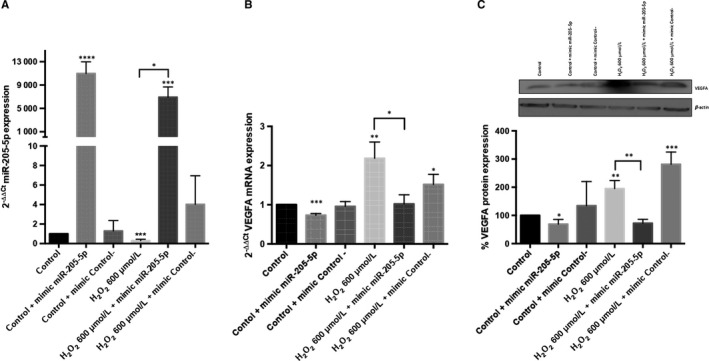
VEGFA expression is regulated by miR‐205‐5p. MiR‐205‐5p expression levels after 48 h of miR‐205‐5p mimic transfection and mimic negative control (A). VEGFA mRNA expression levels after 48 h of transfection with miR‐205‐5p mimic and mimic negative control were measured by qRT‐PCR (B). Western blot analysis for VEGFA protein after miR‐205‐5p mimic and mimic negative control transfection (C). Values are expressed as mean ± SEM (n = 3). Statistically significant differences were set at *P < .05, **P < .01, ***P < .001 and ****P < .0001

In order to test the role of miR‐205‐5p on VEGFA mRNA expression, ARPE‐19 cells were transfected with a miR‐205‐5p mimic. The transfection process did not affect the ARPE‐19 cells viability (File S2). The transfection with a negative control did not affect miR‐205‐5p expression. Transfected ARPE‐19 cells with the mimic, increased miR‐205‐5p levels (Figure 4A), decreasing VEGFA mRNA levels (Figure 4B). Similarly, transfected ARPE‐19 cells also decreased VEGFA mRNA levels after H_2_O_2_ exposure (Figure 4B). These results were confirmed by Western blotting analysis, showing a significant decrease of VEGFA protein expression after miR‐205‐5p mimic transfection in H_2_O_2_ treated cells (Figure 4C). MiR‐205‐5p was inhibited in untreated ARPE‐19 cells and expression of mRNA VEGFA studied. No significant changes were found (File S3).

### 3.5 Vasculogenesis is affected by miR‐205‐5p deregulation via VEGFA

**Figure 5:**
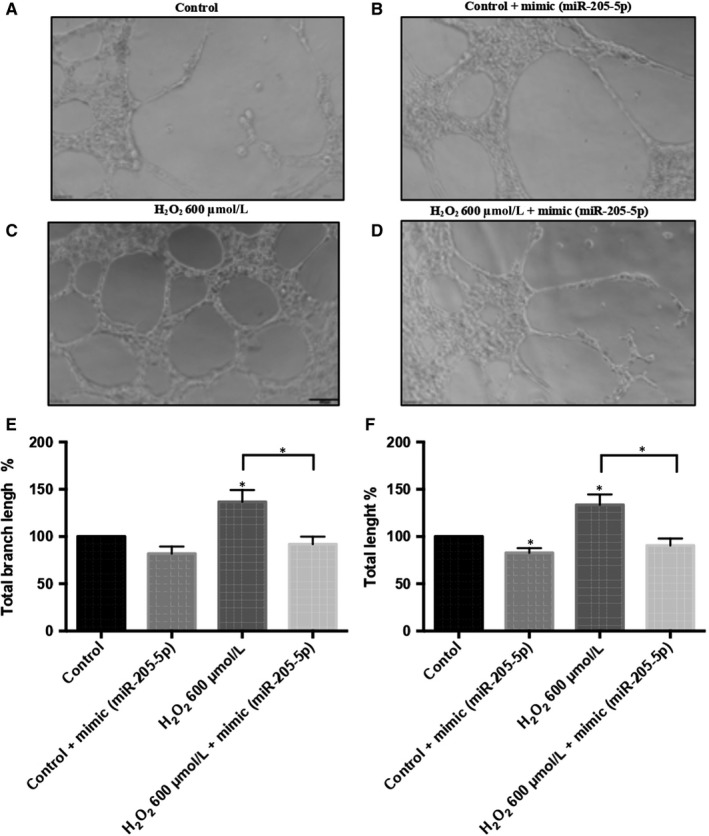
VEGFA‐mediated Vasculogenesis is regulated by miR‐205‐5p. HUVEC cell tube formation under different ARPE‐19 cell culture media conditions. ARPE‐19 control medium (A), mimic(miR‐205‐5p)‐treated ARPE‐19 control (B), H_2_O_2_ 600 µmol/L treated ARPE‐19 (C) and mimic‐treated ARPE‐19 + H_2_O_2_ 600 µmol/L medium (D). Scale bar 100 µmol/L. Total branch length (E) and total length (F) were calculated. Values are expressed as mean ± SEM (n = 3). Statistically significant differences were set at *P < .05

A functional vasculogenesis assay was performed to confirm the inhibiting effect of miR‐205‐5p on VEGFA. HUVEC cells, grown with cell culture media obtained from control ARPE‐19 cells, produced few vascular processes (Figure 5A, 5E and 5F). However, culture media obtained from H_2_O_2_ treated ARPE‐19 cells significantly induced angiogenic changes in HUVEC cells (Figure 5C, 5E and 5F). Interestingly, HUVEC cells grown with mimic‐transfected ARPE‐19 cell culture media significantly abolished these angiogenic changes (Figure 5D, 5E and 5F). Cell culture media from mimic‐transfected ARPE‐19 cells also reduced angiogenesis in control HUVEC cells (Figure 5B, 5E and 5F).

## 4 DISCUSSION

In a previous report, in which miR‐205‐5p was mapped in the mammalian eye, it was found in the epidermis and the anterior segmental epithelia and was not found in RPE.^42^ Other studies have found that miR‐125b^43^ and miR‐23a^44^ are overexpressed in oxidative stress‐induced RPE cells.^41^ This current report is the first one indicating the presence of miR‐205‐5p in ARPE‐19 cells as an RPE cell type. In contrast to the relevance of this miRNA on glioblastoma^13^ or hepatocellular carcinoma,^45^ little information is available about the presence of miR‐205‐5p in the eye.

### 4.1 Oxidative stress down‐regulates miR‐205‐5p expression

As mentioned above, H_2_O_2_ exposure resulted in a significant decrease in miR‐205‐5p levels in ARPE‐19 cells. It has been suggested that miR‐205‐5p positively regulates the peroxiredoxin 2 (PRDX2) pathway.^34^ PRDX2 reduces ROS derived from H_2_O_2_ transformation. In addition, miR‐205‐5p was identified as protective against OS via EGLN2 suppression.^46^ Once considering this, reducing miR‐205‐5p levels after oxidative challenge is opposed to this proposed antioxidant mechanism. Perhaps excessive OS challenge (24 hours H_2_O_2_ 600 µmol/L) leads to a different scenario, far from the physiological conditions that occur in wet AMD or cancer. Future research must address the antioxidant role of miR‐205‐5p. Another relevant finding is that NAC normalized those decreased miR‐205‐5p levels, indicating that miR‐205‐5p is sensitive to ROS.

### 4.2 VEGFA mRNA as a miR‐205‐5p target

According to Yue et al., VEGFA 3’UTR‐mRNA is one of the putative targets of miR‐205‐5p.^7^ In addition, we have identified some potential targets of miR‐205‐5p and their biological processes. Among the potential mRNAs regulated by miR‐205‐5p, 3.5% are angiogenic and 1.3% are related to endothelial cell migration (Figure 2). In fact, it is well documented that miR‐205‐5p acts as a tumour suppressor by regulating cell migration and proliferation.^7^, ^11^, ^12^, ^13^, ^47^ Moreover, it has been suggested that miR‐205‐5p could interact indirectly via PI3K/AKT also involved in angiogenesis.^13^ In the present report, we have observed that VEGFA and miR‐205‐5p are inversely expressed (Figure 3), inducing angiogenesis.

The results shown here confirm the fact that OS increases VEGFA expression levels.^20^, ^48^, ^49^ On the one hand, H_2_O_2_ induction significantly increased both VEGFA mRNA and protein expression in ARPE‐19. On the other hand, the addition of NAC normalized H_2_O_2_‐induced ROS and VEGFA levels. This double result matches with the normalized miR‐205‐5p levels found after NAC exposure. NAC is a precursor of l‐cysteine with scavenging properties and antioxidant effects, interacting with the antioxidant glutathione system.^50^, ^51^ In addition to direct VEGFA regulation, miR‐205‐5p also indirectly regulates ERK1/2 and PI3K/AKT pathways.^13^, ^52^ The PI3K/AKT signalling pathway induces the Nrf2/ARE antioxidant pathway,^53^ and miR‐205‐5p could be involved in this route. Future research should be focused on the antioxidant machinery regulated by miR‐205‐5p.

VEGFA mRNA expression levels negatively correlated with miR‐205‐5p expression, highlighting the transcriptional regulation of VEGFA mRNA by this miRNA. Previous studies have already identified the regulation of VEGFA by miR‐205‐5p.^11^, ^12^, ^13^ Additionally, the results reported here indicate that the VEGFA/miR‐205‐5p correlation might be regulated by OS in ARPE‐19 cells.

Previous data have shown how different oxidative stimuli (ethanol 20 and high glucose^54^) promoted angiogenesis in ARPE‐19 cells. More concretely, OS increased extracellular vesicles released from ARPE‐19 cells, promoting these vascular changes.^20^, ^31^ Moreover, in this report, we demonstrated the effect of miR‐205‐5p/VEGFA regulation on vasculogenic processes induced by oxidative stimuli. OS is a common factor on DR and AMD, supporting the possibility that oxidative challenges on RPE lead to proliferative vascular responses found in AMD and DR.

Anti‐VEGF administration is currently used to control neovascular processes in both DR and wet AMD.^37^ Similarly, anti‐VEGF therapies are also helpful against different forms of cancer, such as glioblastoma and colorectal cancer.^55^ In agreement with this, miR‐205‐5p suppresses VEGFA expression in glioblastoma 7 and also inhibits the PI3K/AKT pathway.^13^ In accordance with this, reducing VEGF‐angiogenesis and PI3K/AKT could decrease tumour cell viability and proliferation. Thus, miR‐205‐5p could be considered as a plausible target against local vascular proliferative retinal processes. Moreover, increasing miR205‐5p levels could be beneficial for these ocular proliferative disorders not only by the direct anti‐VEGFA blockade but also by the PI3K/AKT pathway interaction.

## CONFLICT OF INTEREST

The authors have no conflicts of interest to declare.

## AUTHOR CONTRIBUTIONS

JSP and JMB designed the research programme. MO, LVG and RM performed the research. MO analysed the data. MO, JSP and JMB wrote the paper.

## Data Availability

The data that support the findings of this study are available from the corresponding author upon reasonable request.
