# Supplementary material for: Oxidative stress‐induced angiogenesis is mediated by miR‐205‐5p
Source: J Cell Mol Med. 2019 Dec 21;24(2):1428–36. doi: 10.1111/jcmm.14822 (PMC6991635; doi:10.1111/jcmm.14822)
Supplement: Supplementary file 1 [file JCMM-24-1428-s001.docx]

**Supplementary file 1.** mRNA targets of miR-205-5p related to vascular processes.

| **Abbreviation** | **Name** | **Function** |
| --- | --- | --- |
| RORA | RAR Related Orphan Receptor A | pro-angiogenic[1] |
| MED1 | Mediator of RNA polymerase II transcription subunit 1 | unknown |
| QKI | Protein quaking | pro-angiogenic[2] |
| GATA3 | Trans-ating T-cell-specific transcription factor GATA-3 | anti-angiogenic[3] |
| AGO1 | Protein argonaute-1 | pro-angiogenic[4] |
| INSR | Insulin receptor | pro-angiogenic[5] |
| PLET1 | Placenta-expressed transcript 1 protein | unknown |
| ROCK2 | Rho-associated protein kinase 2 | pro-angiogenic[6] |
| HMGB1 | High mobility group protein B1 | pro-angiogenic[7] |
| ARID1A | AT-rich interactive domain-containing protein 1A | anti-angiogenesis[8] |
| RUNX1 | Runt-related transcription factor 1 | pro-angiogenic[9] |
| PTEN | Phosphatidylinositol 3,4,5-trisphosphate 3-phosphatase and dual-specificity protein phosphatase PTEN | pro-angiogenic[10] |
| ANGPT2 | Angioprotein-2 | pro-angiogenic[11] |
| ERBB4 | Receptor tyrosine-protein kinase erbB-4 | pro-angiogenic[12] |
| MAPK3 | Mitogen-activated protein kinase 3 | pro-angiogenic[13] |
| PRKCE | Protein kinase C epsilon type | anti-angiogenic[14] |
| PRKCA | Protein kinase C alpha type | anti-angiogenic[15] |
| S1PR1 | Sphingosine 1-phosphate receptor 1 | pro-angiogenic[16] |
| LYN | Tyrosine-protein kinase Lyn | pro-angiogenic[17] |
| CSF1 | Macrophage colony-stimulating factor 1 receptor | pro-angiogenic[18] |
| LPAR1 | Lysophosphatidic acid receptor 1 | pro-angiogenic[19] |
| SMAD1 | Mothers against decapentaplegic homolog 1 | pro-angiogenic[20] |
| YAP1 | Transcriptional coactivator YAP1 | pro-angiogenic[21]* |
| AMOT | Angiomotin | anti-angiogenic[22] |
| THBS1 | Thrombospondin-1 | anti-angiogenic[23]* |
| EREG | Proepiregulin | pro-angiogenic[24] |
| VEGFA | Vascular endothelial growth factor-A | pro-angiogenic[25] |
| NRCAM | Neuronal cell adhesion molecule | pro-angiogenic[26] |
| PTPRJ | Receptor-type tyrosine-protein phosphatase eta | pro-angiogenic[27] |
| CALCRL | Calcitonin gene-related peptide type 1 receptor | pro-angiogenic[28] |
| RNF213 | E3 ubiquitin-protein ligase RNF213 | anti-angiogenic[29] |
| CTH | Cystathionine gamma-lyase | pro-angiogenic[30] |

**REFERENCES:**

[1] Sun Y, Liu C-H, Wang Z, Meng SS, Burnim SB, SanGiovanni JP, et al. RORα modulates semaphorin 3E transcription and neurovascular interaction in pathological retinal angiogenesis. FASEB J 2017;31:4492–502. doi:10.1096/fj.201700172R.

[2] Cochrane A, Kelaini S, Tsifaki M, Bojdo J, Vilà-González M, Drehmer D, et al. Quaking Is a Key Regulator of Endothelial Cell Differentiation, Neovascularization, and Angiogenesis. Stem Cells 2017;35:952–66. doi:10.1002/stem.2594.

[3] Asselin-Labat M-L, Sutherland KD, Vaillant F, Gyorki DE, Wu D, Holroyd S, et al. Gata-3 Negatively Regulates the Tumor-Initiating Capacity of Mammary Luminal Progenitor Cells and Targets the Putative Tumor Suppressor Caspase-14. Mol Cell Biol 2011;31:4609–22. doi:10.1128/MCB.05766-11.

[4] Zhao C, Popel AS. Computational Model of MicroRNA Control of HIF-VEGF Pathway: Insights into the Pathophysiology of Ischemic Vascular Disease and Cancer. PLOS Comput Biol 2015;11:e1004612. doi:10.1371/journal.pcbi.1004612.

[5] Heidegger I, Kern J, Ofer P, Klocker H, Massoner P. Oncogenic functions of IGF1R and INSR in prostate cancer include enhanced tumor growth, cell migration and angiogenesis. Oncotarget 2014;5:2723–35. doi:10.18632/oncotarget.1884.

[6] Tagashira T, Fukuda T, Miyata M, Nakamura K, Fujita H, Takai Y, et al. Afadin Facilitates Vascular Endothelial Growth Factor–Induced Network Formation and Migration of Vascular Endothelial Cells by Inactivating Rho-Associated Kinase Through ArhGAP29. Arterioscler Thromb Vasc Biol 2018;38:1159–69. doi:10.1161/ATVBAHA.118.310991.

[7] Nishihiro S, Hishikawa T, Hiramatsu M, Kidani N, Takahashi Y, Murai S, et al. High-Mobility Group Box-1-Induced Angiogenesis After Indirect Bypass Surgery in a Chronic Cerebral Hypoperfusion Model. NeuroMolecular Med 2019. doi:10.1007/s12017-019-08541-x.

[8] Hu C, Li W, Tian F, Jiang K, Liu X, Cen J, et al. Arid1a regulates response to anti-angiogenic therapy in advanced hepatocellular carcinoma. J Hepatol 2018;68:465–75. doi:10.1016/j.jhep.2017.10.028.

[9] Lam JD, Oh DJ, Wong LL, Amarnani D, Park-Windhol C, Sanchez A V., et al. Identification of RUNX1 as a Mediator of Aberrant Retinal Angiogenesis. Diabetes 2017;66:1950–6. doi:10.2337/db16-1035.

[10] Zhang Y, Chen Z, Feng L, Jiang P, Li X, Wang X. Ionizing Radiation-inducible microRNA-21 Induces Angiogenesis by Directly Targeting PTEN. Asian Pacific J Cancer Prev 2019;20:1587–93. doi:10.31557/APJCP.2019.20.5.1587.

[11] Ren L, Wei C, Li K, Lu Z. LncRNA MALAT1 up-regulates VEGF-A and ANGPT2 to promote angiogenesis in brain microvascular endothelial cells against oxygen–glucose deprivation via targetting *miR-145*. Biosci Rep 2019;39:BSR20180226. doi:10.1042/BSR20180226.

[12] Russell KS, Stern DF, Polverini PJ, Bender JR. Neuregulin activation of ErbB receptors in vascular endothelium leads to angiogenesis. Am J Physiol Circ Physiol 1999;277:H2205–11. doi:10.1152/ajpheart.1999.277.6.H2205.

[13] Chen D-B, Feng L, Hodges JK, Lechuga TJ, Zhang H. Human trophoblast-derived hydrogen sulfide stimulates placental artery endothelial cell angiogenesis†. Biol Reprod 2017;97:478–89. doi:10.1093/biolre/iox105.

[14] Hafeez BB, Zhong W, Weichert J, Dreckschmidt NE, Jamal MS, Verma AK. Genetic Ablation of PKC Epsilon Inhibits Prostate Cancer Development and Metastasis in Transgenic Mouse Model of Prostate Adenocarcinoma. Cancer Res 2011;71:2318–27. doi:10.1158/0008-5472.CAN-10-4170.

[15] Mountain DJH, Singh M, Singh K. Downregulation of VEGF-D expression by interleukin-1β in cardiac microvascular endothelial cells is mediated by MAPKs and PKCα/β1. J Cell Physiol 2008;215:337–43. doi:10.1002/jcp.21315.

[16] Liu S, Ni C, Zhang D, Sun H, Dong X, Che N, et al. S1PR1 regulates the switch of two angiogenic modes by VE-cadherin phosphorylation in breast cancer. Cell Death Dis 2019;10:200. doi:10.1038/s41419-019-1411-x.

[17] Hang T-C, Tedford NC, Reddy RJ, Rimchala T, Wells A, White FM, et al. Vascular Endothelial Growth Factor (VEGF) and Platelet (PF-4) Factor 4 Inputs Modulate Human Microvascular Endothelial Signaling in a Three-Dimensional Matrix Migration Context* □ S 2013. doi:10.1074/mcp.M113.030528.

[18] Zheng Q, Li X, Cheng X, Cui T, Zhuo Y, Ma W, et al. Granulocyte-macrophage colony-stimulating factor increases tumor growth and angiogenesis directly by promoting endothelial cell function and indirectly by enhancing the mobilization and recruitment of proangiogenic granulocytes. Tumor Biol 2017;39:101042831769223. doi:10.1177/1010428317692232.

[19] Kanehira M, Fujiwara T, Nakajima S, Okitsu Y, Onishi Y, Fukuhara N, et al. A Lysophosphatidic Acid Receptors 1 and 3 Axis Governs Cellular Senescence of Mesenchymal Stromal Cells and Promotes Growth and Vascularization of Multiple Myeloma. Stem Cells 2017;35:739–53. doi:10.1002/stem.2499.

[20] Oh M-K, Kim I-S. Involvement of placental growth factor upregulated via TGF-β1-ALK1-Smad1/5 signaling in prohaptoglobin-induced angiogenesis. PLoS One 2019;14:e0216289. doi:10.1371/journal.pone.0216289.

[21] Han N, Tian W, Yu N, Yu L. YAP1 is required for the angiogenesis in retinal microvascular endothelial cells via the inhibition of MALAT1‐mediated miR‐200b‐3p in high glucose‐induced diabetic retinopathy. J Cell Physiol 2019:jcp.29047. doi:10.1002/jcp.29047.

[22] Dai X, She P, Chi F, Feng Y, Liu H, Jin D, et al. Phosphorylation of angiomotin by Lats1/2 kinases inhibits F-actin binding, cell migration, and angiogenesis. J Biol Chem 2013;288:34041–51. doi:10.1074/jbc.M113.518019.

[23] Foulsham W, Dohlman TH, Mittal SK, Taketani Y, Singh RB, Masli S, et al. Thrombospondin-1 in ocular surface health and disease. Ocul Surf 2019;17:374–83. doi:10.1016/j.jtos.2019.06.001.

[24] Cai Y, Xie K, Wu H, Wu K. Functional suppression of Epiregulin impairs angiogenesis and aggravates left ventricular remodeling by disrupting the extracellular‐signal‐regulated kinase1/2 signaling pathway in rats after acute myocardial infarction. J Cell Physiol 2019;234:jcp.28503. doi:10.1002/jcp.28503.

[25] Yue X, Wang P, Xu J, Zhu Y, Sun G, Pang Q, et al. MicroRNA-205 functions as a tumor suppressor in human glioblastoma cells by targeting VEGF-A. Oncol Rep 2012;27:1200–6. doi:10.3892/or.2011.1588.

[26] Slevin M, Krupinski J, Rovira N, Turu M, Luque A, Baldellou M, et al. Identification of pro-angiogenic markers in blood vessels from stroked-affected brain tissue using laser-capture microdissection. BMC Genomics 2009;10:113. doi:10.1186/1471-2164-10-113.

[27] Fournier P, Dussault S, Fusco A, Rivard A, Royal I. Tyrosine Phosphatase PTPRJ/DEP-1 Is an Essential Promoter of Vascular Permeability, Angiogenesis, and Tumor Progression. Cancer Res 2016;76:5080–91. doi:10.1158/0008-5472.CAN-16-1071.

[28] Dong Y-L, Reddy DM, Green KE, Chauhan MS, Wang H-Q, Nagamani M, et al. Calcitonin Gene-Related Peptide (CALCA) Is a Proangiogenic Growth Factor in the Human Placental Development1. Biol Reprod 2007;76:892–9. doi:10.1095/biolreprod.106.059089.

[29] Ito A, Fujimura M, Niizuma K, Kanoke A, Sakata H, Morita-Fujimura Y, et al. Enhanced post-ischemic angiogenesis in mice lacking RNF213; a susceptibility gene for moyamoya disease. Brain Res 2015;1594:310–20. doi:10.1016/j.brainres.2014.11.014.

[30] van den Born JC, Mencke R, Conroy S, Zeebregts CJ, van Goor H, Hillebrands JL. Cystathionine γ-lyase is expressed in human atherosclerotic plaque microvessels and is involved in micro-angiogenesis. Sci Rep 2016;6:34608. doi:10.1038/srep34608.
