# Supplementary figures and images for: Oxidative stress‐induced angiogenesis is mediated by miR‐205‐5p
Source: J Cell Mol Med. 2019 Dec 21;24(2):1428–36. doi: 10.1111/jcmm.14822 (PMC6991635; doi:10.1111/jcmm.14822)

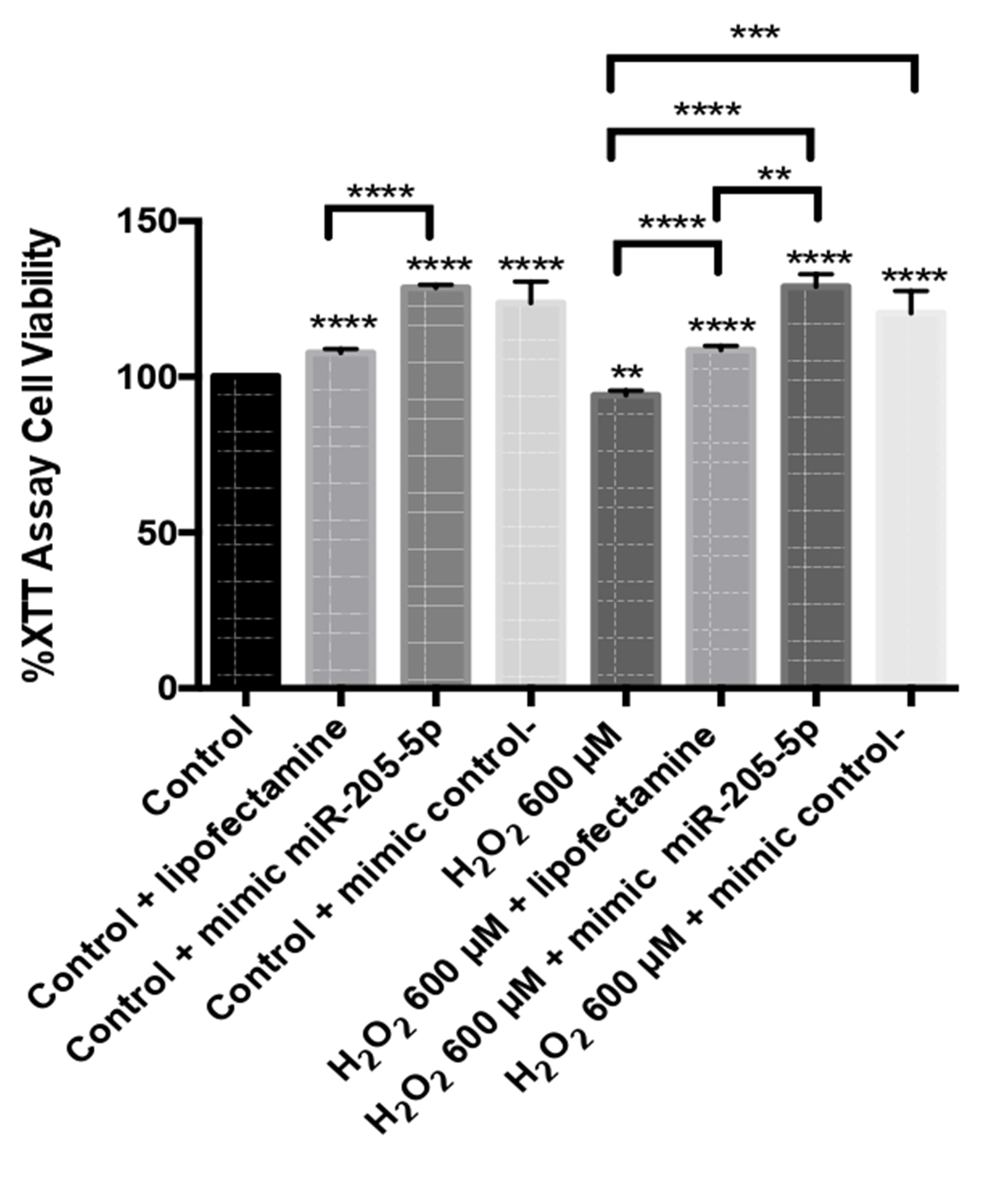

Supplement: Supplementary file 2 [file JCMM-24-1428-s002.tif]

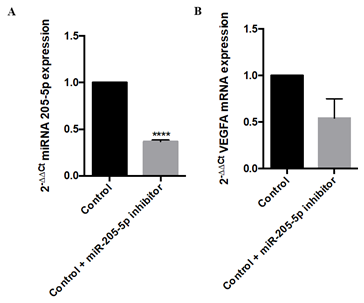

Supplement: Supplementary file 3 [file JCMM-24-1428-s003.tif]
